# Prioritizing medication safety in care of people with cancer: clinicians’ views on main problems and solutions

**DOI:** 10.7189/jogh.07.011001

**Published:** 2017-06

**Authors:** Lorainne Tudor Car, Nikolaos Papachristou, Catherine Urch, Azeem Majeed, Rifat Atun, Josip Car, Charles Vincent

**Affiliations:** 1 Lee Kong Chian School of Medicine, Nanyang Technological University, Singapore; 2 Department of Primary Care and Public Health, School of Public Health, Imperial College London, UK; 3 Department of Global Health and Population, Harvard T.H. Chan School of Public Health, Harvard, Boston, Massachusetts, USA; 4 Imperial College Healthcare NHS Trust, ‎St Mary’s Hospital, London, UK; 5 Health Services and Outcomes Research Programme, Lee Kong Chian School of Medicine, Nanyang Technological University, Singapore; 6 Department of Experimental Psychology, Medical Sciences Division, University of Oxford, UK

## Abstract

**Background:**

Cancer care is liable to medication errors due to the complex nature of cancer treatment, the common presence of comorbidities and the involvement of a number of clinicians in cancer care. While the frequency of medication errors in cancer care has been reported, little is known about their causal factors and effective prevention strategies. With a unique insight into the main safety issues in cancer treatment, frontline staff can help close this gap. In this study, we aimed to identify medication safety priorities in cancer patient care according to clinicians in North West London using PRIORITIZE, a novel priority–setting approach.

**Methods:**

The project steering group determined the scope, the context and the criteria for prioritization. We then invited North West London cancer care clinicians to identify and prioritize main causes for, and solutions to, medication errors in cancer care. Forty cancer care providers submitted their suggestions which were thematically synthesized into a composite list of 20 distinct problems and 22 solutions. A group of 26 clinicians from the initial cohort ranked the composite list of suggestions using predetermined criteria.

**Results:**

The top ranked problems focused on patients’ poor understanding of treatments due to language or education difficulties, clinicians’ insufficient attention to patients’ psychological distress, and inadequate information sharing among health care providers. The top ranked solutions were provision of guidance to patients and their carers on what to do when unwell, pre–chemotherapy work–up for all patients and better staff training. Overall, clinicians considered improved communication between health care providers, quality assurance procedures (during prescription and monitoring stages) and patient education as key strategies for improving cancer medication safety. Prescribing stage was identified as the most vulnerable to medication safety threats. The highest ranked suggestions received the strongest agreement among the clinicians.

**Conclusions:**

Clinician–identified priorities for reducing medication errors in cancer care addressed various aspects of cancer treatment. Our findings open up an opportunity to assess the congruence between health care professional suggestions, currently implemented patient safety policies and evidence base.

Medication errors, defined as preventable events that may lead to inappropriate medication use or patient harm, are a serious and common threat to cancer patients [1,2]. In an oncology outpatient department in the US, medication errors occurred in 7% of adults and 19% of children [3]. A systematic review reported that approximately 20% of palliative cancer patients were prescribed potentially inappropriate medications [4]. Cancer treatment is highly predisposed to errors due to its multifaceted and dynamic nature. Chemotherapy, consisting of complex regimens of potent and potentially toxic drugs, has to be administered repeatedly, according to protocols and frequently adapted to patients’ conditions. This is coupled with a considerable burden of concurrent illnesses, a common need for supportive therapy and the involvement of many different clinicians in provision of care [5–7].

The evidence on cancer medication safety, ie, freedom from preventable harm with medication use, mostly focuses on rates and types of medication errors in specific forms of chemotherapy or cancers [8]. It includes analysis of patient medical records, incident reports and prospective observational studies [9–11]. Little is known about the main causal factors to cancer medication errors and the specific interventions that could lead to significant improvements in safety.

One way of addressing this lack of evidence is by exploring clinicians’ unique insight into the safety and quality of cancer treatment [12]. Cancer care clinicians offer an important source to guide our understanding of the cancer safety issues which has not to date been routinely and formally drawn on. In this study, we aimed to identify priorities for medication safety in care of people with cancer according to cancer care clinicians in North West London.

## METHODS

### PRIORITIZE and the study scope

**Figure 1:**
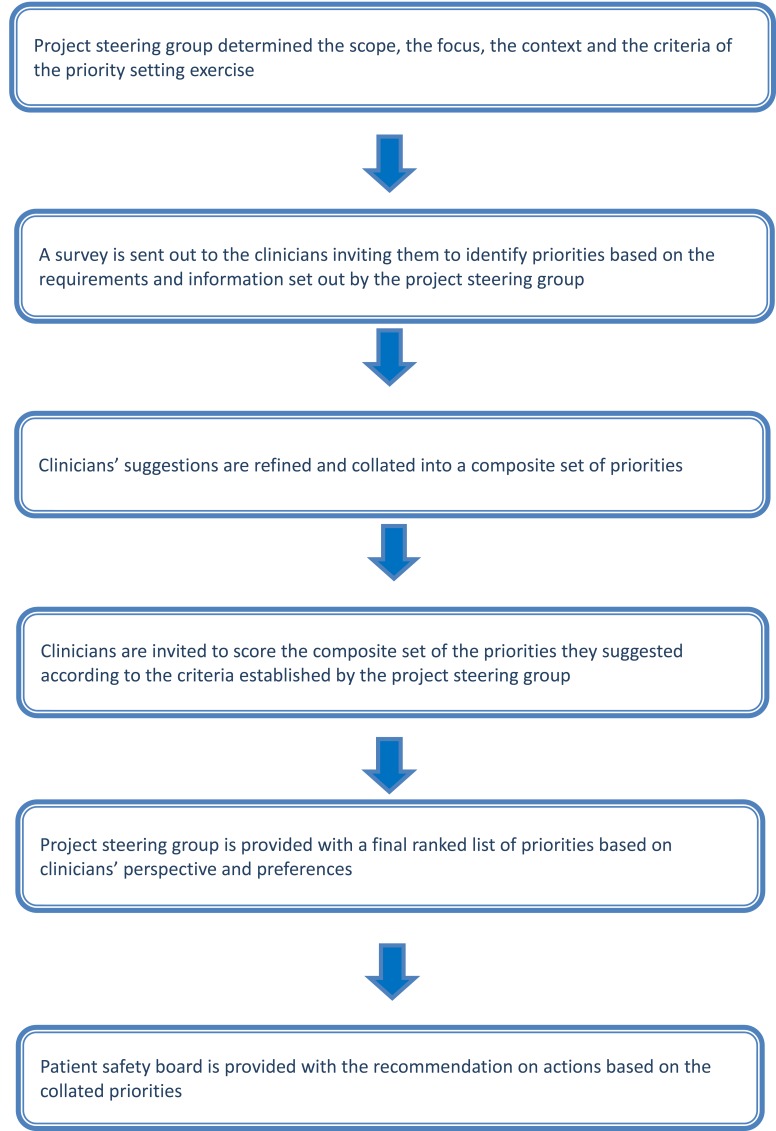
PRIORITIZE methodology flow diagram.

We developed and implemented PRIORITIZE, an adaptation of Child Health and Nutrition Research Initiative (CHNRI) approach, to determine the main problems and solutions relating to medication safety in cancer care (**Figure 1**). The CHNRI methodology has been used extensively to inform policymakers, funding bodies and international organizations about priorities for research [13–15]. PRIORITIZE focuses on priorities in health care services delivery using clinicians’ as experts and determines priorities using two corresponding viewpoints: problems and solutions. The final output of this approach is presentation of the top priorities categorized according to level for the implementation: a) actions for clinicians b) actions for health care organisations and hospitals and c) actions for health system custodians. As this study was deemed a service evaluation and an innovative quality and safety improvement initiative, it did not require ethics or governance approval [16,17]. During the study’s first stage, the project steering group (Imperial College Health Partners), decided to focus on two topics relating to cancer care patient safety: medication safety and delayed diagnosis (presented elsewhere) [18]. Imperial College Health Partners is an organization that unifies NHS health care providers, clinical commissioning groups and leading universities across North West London with the aim of improving quality of health care delivery [19]. The steering group also chose the criteria to guide prioritisation of collated suggestions, ie, scoring of problems and solutions (**Box 1**).

Box 1 **Scoring criteria for prioritization of collated suggestions**

For problems:

- Frequency – This patient safety threat is common.
- Severity – This patient safety threat leads to high rates of mortality, morbidity and incapacity.
- Inequity – This patient safety threat affects lower socio–economic groups or ethnic minorities more than other groups.
- Economic impact – The consequences of this patient safety threat are costly to the healthcare system.
- Responsiveness to solution – This incident is amenable to a solution within 5 years.

For solutions:

- Feasibility – The implementation of this solution is feasible.
- Cost–effectiveness – This solution is cost–effective.
- Potential for saving lives – This solution would save lives.

### Identifying cancer medication safety priorities

We developed an open–ended questionnaire for clinicians to identify the main problems and solutions relating to medication safety in cancer care. It was piloted on a smaller sample of four primary care physicians and trainees recruited through our Department and amended based on the received feedback (see Appendix S1 in **Online Supplementary Document(Online Supplementary Document)**). The questionnaire was distributed in a paper–based and an equivalent online version and disseminated via email lists and snowballing (participants were asked to forward the survey to colleagues). We targeted oncology consultants, general practitioners, trainees, nurses and pharmacists.

### Scoring of cancer medication safety priorities

The collected suggestions were examined using content analysis with open coding to categorise the free–text responses. Suggestions which were sufficiently similar were combined. In the second phase, we asked clinicians to categorize the suggestions using the predetermined scoring criteria and four options: 1 for “Yes – I agree with the statement”, 0 for “No – I do not agree with the statement”, 0.5 for “Unsure – I am unsure whether or not I agree” and blank (no response) for “Unaware – I do not feel sufficiently familiar or confident to score this suggestion” (see Appendix S2 in **Online Supplementary Document(Online Supplementary Document)**). As the scoring was time demanding (an average 1 hour to complete), we offered a token payment to the participants in a form of a £50 voucher. Clinicians who performed scoring of the priorities were arbitrarily selected from the initial cohort of cancer care clinicians.

### Computation of priority scores and average expert agreement

**Figure 2:**
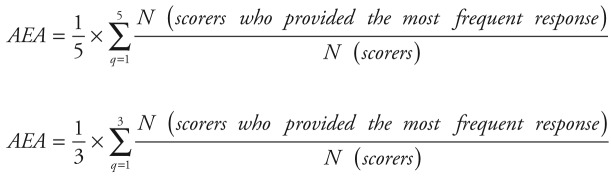
Formula for calculating average expert agreement; q is a question that experts are being asked to evaluate competing patient safety threats.

The data from the scoring sheet was collected and analyzed with SPSS (v. 21), IBM, New York, USA. We calculated the intermediate scores (ie, scores for each criterion for every suggestion) by adding up all the answers (“1,” “0” or “0.5”) and dividing the sum by the number of received answers. Intermediate scores for suggestions were therefore assigned a value between 0 to 100. The overall priority score for every suggestion was then computed as the mean of the scores for each criterion (ie, five criteria for problems and three for solutions). Suggestions that were ranked higher received more “Yes” responses for each of the criteria and a higher overall score. Kappa statistics was deemed an inappropriate test to determine inter–rater agreement in this study due to the sample size, the non–standardised categorical nature of data, the option of blank response to some statements and the number of our different criteria used for scoring. Instead, we evaluated inter–rater agreement using the average expert agreement (AEA) (**Figure 2**) [13]. AEA is the share of scorers selecting the most common score for each research question and indicates the degree of clinicians’ agreement on priorities. AEA was calculated using the formula in **Figure 2**.

We classified the collated suggestions for medication safety in cancer care using an adapted model of medication delivery and the London Protocol, a framework for aa comprehensive investigation and analysis of patient safety incident, for use by clinicians, risk and patient safety managers, researchers and others wishing to reflect and learn from clinical incidents [20,21] (see Appendix S3 in **Online Supplementary Document(Online Supplementary Document)**).

## RESULTS

**Figure 3:**
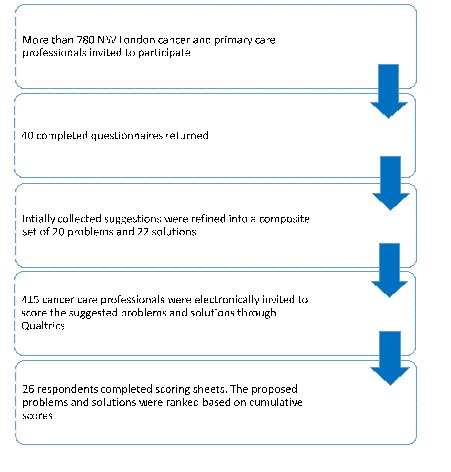
Participants’ flow diagram.

In the first phase we invited around 780 cancer care clinicians and received 40 completed questionnaires with the majority by oncology consultants (n = 15, 37.5%) and specialty trainees (n = 15, 37.5%) (see Appendix S4 in **Online Supplementary Document(Online Supplementary Document)**). We collated 101 problems and 53 solutions relating to cancer medication safety and thematically merged them into 20 distinct problems and 22 solutions. From the phase 1 cohort, 415 cancer care clinicians were invited to score the composite list of suggestions resulting in 26 fully completed scoring sheets (**Figure 3**).

The top ranked problems leading to medication errors in cancer treatment according to clinicians are patients’ poor understanding of treatments due to language or education difficulties, insufficient attention to psychological distress or illness and clinicians’ lack of access to information on treatments administered in other hospitals or by other health care providers (**Table 1**). The top three solutions to medication safety threats are guidance to patients and their carers on what to do when unwell, an appropriate pre–chemotherapy work up for all patients and better training of staff. Clinicians identified prescribing stage as the most vulnerable to medication safety threats (**Table 2**).

**Table 1:** Top ten solutions to medication-related problems in cancer care*

Rank	Proposed medication–related problems in cancer care	Total Priority Score	Breakdown point in the medication process	Contributor factor
1	Patients with poor understanding of treatments due to language or education difficulties may miss treatments or not understand the importance of reporting side effects leading to worsening of illness	75.5	Administering/monitoring	Patient
2	Insufficient attention to recognizing and managing serious psychological distress or illness due to oncological problem and treatment leads to non–compliance and/or worsening of patient’s condition	66	Monitoring	Individual staff
3	Inability to obtain information on treatments given in other hospitals or by other health care providers eg, palliative care team mean that the oncology team may administer inappropriate treatments or delay treatment while waiting for the information	62.5	Administering	Task design
4	Complications of central access lines inserted for chemotherapy lead to patient morbidity or delayed treatments	59.5	Administering	–
5	Patients have difficulty accessing acute oncology services outside of routine hours leading to delayed treatment of side effects or complications with significant negative consequences (eg, preventable hospitalizations)	58	Monitoring	Organisation
6	Toxicity or severe allergic reactions from chemotherapy	55.5	Administering	–
7	Drugs may be stopped for procedures eg, anticoagulants but not restarted leading to adverse events for patients such as thromboembolic events	55	Administering	Individual staff
8	Interactions between medications are not automatically highlighted meaning that inappropriate drugs may be administered together	53.5	Administering	Task design
9	Patients do not inform their oncologist of side effects meaning that the chemotherapy dose is not altered and the side effects become worse	52	Monitoring	Patient
10	Too little information on chemotherapy for patients prior to starting treatment meaning that they do not know or recognize signs of complications or serious illness and who and when to contact	50.5	Prescribing	Patient

*(Clinicians scored problems using the following criteria: frequency, severity, inequity, economic impact and responsiveness to solution (**Box 1**). The scoring options were 1 for “yes (eg, this problem is common)”, 0 for “no (eg, this problem is uncommon)”, 0.5 for “unsure (eg, I am unsure if this problem is common)” and blank for “unaware (eg, I do not know if his problem is common)”. Total Priority score is the mean of scores for each of the five criteria and is ranging from 0 to 100. Higher ranked problems received more “Yes” responses for each of the criteria and a higher score). All tables use clinicians’ verbatim statements which were only exceptionally reworded for clarity.

**Table 2:** Top ten solutions to medication–related problems cancer care*

Rank	Proposed solution for medication–related problems in cancer care	Total Priority Score	Breakdown point in the medication process	Related defense barrier
1	Provide information for patients and their carers on what to do when unwell eg, card with contact numbers	93.3	Monitoring	Patient
2	All patients should receive an appropriate pre–chemotherapy work up	92.5	Administering	Task design
3	Improve training of staff	91.7	Prescribing, transcribing, dispensing, administering, monitoring	Working environment
4	Develop a checklist for clinicians so that important points in the history or tests are not missed	90.0	Prescribing	Task design
5	Ensure patients have relevant written information for community clinicians to ensure that appropriate treatments are given	89.2	Administering	Patient
6	Enable staff to access patient records remotely so that on call staff are fully aware of the patient’s history	87.5	Prescribing, monitoring	Task design
7	Improve the staff:patient ratios	86.7	Prescribing, transcribing, dispensing, administering, monitoring	Working environment
8	Advise patients to check their temperature regularly to detect sepsis earlier	85.8	Monitoring	Patient
9	Improve communication with pharmacy about drugs and dose adjustments so that delays in drug administration do not occur	85.8	Transcribing	Team
10	Attach the chemotherapy prescription chart to the routine drug chart so drugs are not missed	84.2	Prescribing	Task design
11	Advise patients to contact hospital early in day if unwell to ensure appropriate staff available	84.2	Monitoring	Patient

*(Clinicians scored solutions using feasibility and cost–effectiveness solutions (**Box 1**). The scoring options were 1 for “yes (eg, this solution is feasible)”, 0 for “no (eg, this solution is unfeasible)”, 0.5 for “unsure (eg, I am unsure if this solution is feasible)” and blank for “unaware (eg, I do not know if this solution is feasible)”. Total Priority score is the mean of the scores for each of the two criteria and is ranging from 0 to 100. Higher ranked solutions).

Overall, the proposed problems focused on poor communication among clinicians and with patients; inadequate quality assurance processes; errors during the prescription and monitoring stage and patients’ lack of awareness or poor understanding of chemotherapy (Table S5 in **Online Supplementary Document(Online Supplementary Document)**). Proposed solutions overall focused on improving information integration and communication among health care services, introducing quality assurance interventions during the prescribing and monitoring stage, and enhanced patient empowerment and education (Table S6 in **Online Supplementary Document(Online Supplementary Document)**).

Several of the proposed problems focused on patients’ role in cancer medication safety (Table S5 in **Online Supplementary Document(Online Supplementary Document)**). They included poor understanding of treatments due to language or education difficulties, not informing their oncologist about the side effects, not recognizing complications and not knowing whom to inform, and attending their GP rather than oncology services (Table S5 in **Online Supplementary Document(Online Supplementary Document)**). Correspondingly, patient empowerment and education were highlighted as key safety priorities (Table S6 in **Online Supplementary Document(Online Supplementary Document)**). Pertinent suggestions included tailored guidance on what to do when feeling unwell, having treatment records to ensure administration of appropriate treatment from the community providers, increasing the number of clinical nurse specialists to provide patient education and continuity of care as well as encouraging frequent body temperature checks and increased physical activity.

Clinicians viewed patients from lower socio–economic group as more commonly affected by poor understanding of treatment, clinicians’ inattention to comorbidities and lack of access to information on their treatment from other health care providers. This group of patients was also considered more likely to receive less information on chemotherapy as well as to visit their GP rather than oncology service for complications from chemotherapy leading to delays in treatment or inappropriate advice or treatments (Table S5 in **Online Supplementary Document(Online Supplementary Document)**).

Suggestions that were seen as least important for cancer medication safety overall related to issues with the chemotherapy prescribing system, the need for more frequent blood tests, chemotherapy dose calculation errors and the use of personalised medicine approaches. The top ranked suggestions had the highest AEA, ie, there was a stronger consensus among clinicians for the top suggestions compared to those ranked lower. Proposed solutions received higher AEA scores compared to problems, ie, clinicians agreed more on the ranking of solutions compared to the ranking of problems (Table S5 in **Online Supplementary Document(Online Supplementary Document)**).

## DISCUSSION

In this study, clinicians from North West London identified priorities for improving cancer medication safety. The top ranked problems were patients’ poor understanding of treatments, clinicians’ insufficient attention to patients’ psychological distress and poor information exchange among health care providers. The top ranked solutions were guidance to patients and their carers on what to do when unwell, an appropriate pre–chemotherapy work up for all patients and better staff training. Overall, clinicians considered better communication between health care providers, quality assurance procedures and patient education as key to ensuring cancer medication safety. The highest ranked suggestions received the strongest agreement among the clinicians. Many identified suggestions for cancer medication safety are feasible, affordable and could contribute to improvements to medication safety in cancer care.

We have also used PRIORITZE to identify primary care clinicians’ medication safety priorities in primary care [22]. While the overarching themes were the same (eg, patient education, communication and information sharing across different health care providers and quality assurance procedures), particular priorities differ significantly. Primary care medication safety priorities were broader in scope and included several suggestions relating to transfer of care between different health care providers. Conversely, cancer medication priorities seem more focused and many addressed the need for improved sharing of information and communication with patients.

According to the clinicians in our study, cancer patients lack information about the potential side–effects and who to turn to in case of treatment complications. This was seen as more common in patients from lower socio–economic groups or ethnic minorities. Such lack of guidance is concerning given the essential role patients can have as 'vigilant partners' in prevention of chemotherapy medication errors [20,21]. In educating patients about their cancer treatment, health care professionals should consider the content, structure, delivery mode, potential information overload and a need for message reinforcement [9]. Corresponding solutions in our study included provision of tailored information on what to do and who to call if feeling poorly, instructing patients to check their temperature regularly and to contact hospital early in the day if unwell, encouraging patients to undertake increased physical activity and increasing the number of clinical nurse specialists to improve patient education.

The collated suggestions, while more detailed, correspond in part to the author–nominated list of preventive interventions for medication errors in a US oncology outpatient department [1]. Improved communication, standardized ordering sheet and patient education about home medications have been highlighted in both studies as major safety threats. Furthermore, fragmentation in cancer treatment noted in this study has also been observed in other settings [7,23–25]. A recently published randomized controlled trial on pharmacist–led medication reconciliation intervention, aligned with some of the clinician–identified solutions in our study (eg, enabling remote access to patient records and closer links with pharmacy), showed reduction in the incidence of errors in cancer patient [26]. However, the effectiveness of other collated solutions is unclear as the evidence on effective interventions to reduce medication errors in cancer care is lacking [27].

### Limitations

We recruited a small, self–selected sample, potentially different from the clinicians refusing to take part in this study which may have influenced the generalizability of our findings. The low response rate is common in physician surveys, especially those focusing on emotionally–laden topics and including open–ended questions [28,29]. Furthermore, the number of participants corresponds to those in other priority–setting exercises involving health care professionals or employing the CHNRI methodology [30–32]. While our findings correspond to the existing literature, it is unclear how applicable they are to other settings. Patient safety incidents are often context–specific as reflected in a study on medication errors across different outpatient oncology clinics [3]. The advantage of PRIORITIZE is that allows discovery of local safety priorities and customization of patient safety interventions to the study setting.

In comparison to a standard Delphi approach, in PRIORITIZE the number of discussed suggestions is larger, the contribution of all participants equivalent and the prioritization transparent. Yet, as a novel priority–setting methodology, PRIORITIZE could be further refined and validated. The scoring of the solutions could be streamlined through the development of a platform–agnostic information technology tool. Some problems identified in our study related to chemotherapy–related adverse effects (eg, “toxicity or severe allergic reactions from chemotherapy”) rather than causal factors for safety issues. However, by inviting clinicians to identified both problems and solutions, we managed to capture relevant data. In future, this could be enhanced by providing examples which would guide the specificity of responses. Recent CHNRI–focused validity assessments reveal that, in most cases and under most assumption, the collective knowledge will be more accurate than the knowledge of an “average” individual [32]. It also shows that that the collective opinion of around 50 experts expressed was sufficient to reach steady findings and consensus on rankings [33]. These promising insights could also be verified as part of the PRIORITIZE approach.

### Implications for practice and policy

Using a bottom–up approach with clinicians as change agents, we collated a number of concrete, locally relevant and affordable suggestions on cancer medication safety priorities. The suggestions focused on information integration among cancer care providers, implementation of quality assurance procedures and stronger patient education. Some suggestions correlated (eg, “Inability to obtain information on treatments given in other hospitals or by other healthcare providers” and “Enable staff to access patient records remotely”), reinforcing the importance of certain priorities.

Clinicians often report feeling marginalised in patient safety policy development as well as hesitant toward incident reporting due to lack of anonymity, time and the risk of victimisation [34–36]. The information produced by the incident reporting system has been found to be inaccurate, incomplete and difficult to analyze, making it hard to spot dangerous trends or problem [37,38]. Patient safety analytical approaches such as root cause analysis are unable to detect latent causes of error if health care professionals are uncomfortable with exposing safety weaknesses [39]. PRIORITIZE enables anonymous and structured voicing of safety concerns from a large number of health care providers [40–42]. It corresponds to calls for greater inclusion of health care staff in patient safety research, uncovering of local patient safety priorities and development of solutions to safety issues [43].

Future steps should include comparison of local cancer safety policies, organisational data on cancer medication safety and collated clinician–identified priorities to signpost the type of intervention or research that is needed. There is also a need for robust, experimental studies to help determine effective cancer medication safety strategies and support inclusion of clinician–identified suggestions into safety polices. Finally, PRIORITIZE could be used as a routine patient safety assessment tool to trigger staff’s involvement, evaluate patient safety culture, enable country–wide patient safety comparison and development of locally tailored safety strategies.
