# Supplementary material for: Prioritizing medication safety in care of people with cancer: clinicians’ views on main problems and solutions
Source: J Glob Health. 2017 Jun 10;7(1):011001. doi: 10.7189/jogh.07.011001 (PMC5475313; doi:10.7189/jogh.07.011001)
Supplement: Online Supplementary Document [file jogh-07-011001-s001.pdf]

Online Supplementary Document

Tudor Car et al. Prioritizing medication safety in care of people with cancer: clinicians' views on main problems and solutions

J Glob Health 2017;7:011001

Appendix S1. Initial questionnaire on causes of and solutions to cancer medication safety

IMPERIAL COLLEGE HEALTH PARTNERS

WHO Collaborating Centre for Public Health Education and Training

Patient safety in cancer care

Dear *Colleague*,

This survey aims to capture (1) *your* perspective and (2) *your* solutions for incidents that affect the safety of patients with cancer. It has been designed by the Department of Primary Care and Public Health, Imperial College and Imperial College Health Partners to guide patient safety improvement initiatives in NW London and on a wider scale. The results will be used anonymously.

Please specify your role:

Nurse	Specialist Trainee	Consultant	GP	Social Care Professional	Pharmacist	Other (please specify) ________________
○	○	○	○	○	○	○

1. Chemotherapy

A. Please name **3** main chemotherapy-related complications affecting the safety of people in cancer care.

B. How could we reduce or prevent chemotherapy-related complications affecting the safety of patients in cancer care?

2. Delayed Diagnosis

A. Please name **3** main contributors to a delayed diagnosis of cancer.

B. How could we reduce or prevent a delayed diagnosis of cancer?

3. Keeping an eye on care

How could patient safety be better monitored in a GP surgery or a hospital?

4. What's on your mind?

How could we make the care of people with cancer safer?

Thank you very much for your time and effort.

Your contribution will help improve patient safety in cancer care. For more information and to learn more about the outcomes of the study, please contact Dr. Josip Car at josip.car@imperial.ac.uk

Appendix S2. Scoring questionnaire

WHO Collaborating Centre
for Public Health Education and Training

Patient safety priorities in cancer care

Dear *Colleague,*

Thank you for participating in the first step of this process where you identified patient safety problems in cancer care and proposed solutions. They are all listed below. In this second and final step, please indicate your agreement or disagreement with all the statements in the table below using the following criteria:

Y(es) : if you agree
N(o) : if you disagree
UnS(ure) : if you are aware of the problem, but unsure about the answer
UnA(ware) : if you are not sufficiently aware of the problem

Chemotherapy related problems in cancer care	This patient safety threat is common				This patient safety threat leads to high rates of mortality, morbidity and incapacity				This patient safety threat affects more lower socio-economic groups or ethnic minorities				The consequences of this patient safety threat are costly to the healthcare system				This incident is amenable to a solution within 5 years			
	Y	N	UnS	UnA	Y	N	UnS	UnA	Y	N	UnS	UnA	Y	N	UnS	UnA	Y	N	UnS	UnA
1. The chemotherapy prescribing system (e.g. ARIA) is separate from the prescribing system for other drugs leading to drug doses and drug interactions being missed	○	○	○	○	○	○	○	○	○	○	○	○	○	○	○	○	○	○	○	○
2. The chemotherapy prescribing system (e.g. ARIA) is difficult to use leading to drug dosing and timing errors	○	○	○	○	○	○	○	○	○	○	○	○	○	○	○	○	○	○	○	○
3. The chemotherapy prescribing system (e.g. ARIA) does not automatically carry forward dose adjustments in regimens leading to inappropriate dosing of chemotherapy drugs	○	○	○	○	○	○	○	○	○	○	○	○	○	○	○	○	○	○	○	○
4. The chemotherapy prescribing system (e.g. ARIA) does not automatically change the dates when chemotherapy is due if one dose is delayed leading to administration of chemotherapy drugs at the wrong time in the cycle	○	○	○	○	○	○	○	○	○	○	○	○	○	○	○	○	○	○	○	○
5. Pharmacists spend significant amounts of time correcting errors on the chemotherapy prescribing system (e.g. ARIA) leading to delays in administration of drugs	○	○	○	○	○	○	○	○	○	○	○	○	○	○	○	○	○	○	○	○
6. Drugs may be stopped for procedures e.g. anticoagulants but not restarted leading to adverse events for patients such as thromboembolic events	○	○	○	○	○	○	○	○	○	○	○	○	○	○	○	○	○	○	○	○
7. Incorrect doses of chemotherapy are administered due to inaccurate calculations of doses	○	○	○	○	○	○	○	○	○	○	○	○	○	○	○	○	○	○	○	○
8. Toxicity or severe allergic reactions from chemotherapy	○	○	○	○	○	○	○	○	○	○	○	○	○	○	○	○	○	○	○	○
9. Interactions between medications are not automatically highlighted meaning that inappropriate drugs may be administered together	○	○	○	○	○	○	○	○	○	○	○	○	○	○	○	○	○	○	○	○

Chemotherapy related problems in cancer care	This patient safety threat is common				This patient safety threat leads to high rates of mortality, morbidity and incapacity				This patient safety threat affects more lower socio-economic groups or ethnic minorities				The consequences of this patient safety threat are costly to the healthcare system				This incident is amenable to a solution within 5 years			
	Y	N	UnS	UnA	Y	N	UnS	UnA	Y	N	UnS	UnA	Y	N	UnS	UnA	Y	N	UnS	UnA
10. Inappropriate antibiotics are administered for sepsis	○	○	○	○	○	○	○	○	○	○	○	○	○	○	○	○	○	○	○	○
11. Issues such as anaemia, thrombocytopenia or electrolyte disturbance from chemotherapy are not detected due to insufficient monitoring	○	○	○	○	○	○	○	○	○	○	○	○	○	○	○	○	○	○	○	○
12. Too little information on chemotherapy for patients prior to starting treatment meaning that they do not know or recognize signs of complications or serious illness and who and when to contact	○	○	○	○	○	○	○	○	○	○	○	○	○	○	○	○	○	○	○	○
13. Patients attend their GP rather than oncology service for complications from chemotherapy which results in delays in treatment or inappropriate advice or treatments	○	○	○	○	○	○	○	○	○	○	○	○	○	○	○	○	○	○	○	○
14. Patients have difficulty accessing acute oncology services outside of routine hours leading to delayed treatment of side effects or complications with significant negative consequences (e.g. preventable hospitalizations)	○	○	○	○	○	○	○	○	○	○	○	○	○	○	○	○	○	○	○	○
15. Patients are not weighed on each cycle of chemotherapy leading to incorrect doses of chemotherapy on different cycles	○	○	○	○	○	○	○	○	○	○	○	○	○	○	○	○	○	○	○	○
16. Patients do not inform their oncologist of side effects meaning that the chemotherapy dose is not altered and the side effects become worse	○	○	○	○	○	○	○	○	○	○	○	○	○	○	○	○	○	○	○	○
17. Insufficient attention to recognising and managing serious psychological distress or illness due to oncological problem and treatment leads to non-compliance and/or worsening of patient's condition	○	○	○	○	○	○	○	○	○	○	○	○	○	○	○	○	○	○	○	○
18. Inability to obtain information on treatments given in other hospitals or by other healthcare providers e.g. palliative care team mean that the oncology team may administer inappropriate treatments or delay treatment while waiting for the information	○	○	○	○	○	○	○	○	○	○	○	○	○	○	○	○	○	○	○	○
19. Patients with poor understanding of treatments due to language or education difficulties may miss treatments or not understand the importance of reporting side effects leading to worsening of illness	○	○	○	○	○	○	○	○	○	○	○	○	○	○	○	○	○	○	○	○
20. Complications of central access lines inserted for chemotherapy lead to patient morbidity or delayed treatments	○	○	○	○	○	○	○	○	○	○	○	○	○	○	○	○	○	○	○	○

Please add any further ideas or comments that you believe are important and should be included in this study.

Y(es) : if you agree
N(o) : if you disagree
UnS(ure) : if you are aware of the problem, but unsure about the answer
UnA(ware) : if you are not sufficiently aware of the problem

Suggested solutions to chemotherapy related problems in cancer care	This solution is cost-effective				The implementation of this solution is feasible				This solution would save lives			
	Y	N	UnS	UnA	Y	N	UnS	UnA	Y	N	UnS	UnA
1. Improve training of staff	○	○	○	○	○	○	○	○	○	○	○	○
2. All patients should receive an appropriate pre-chemotherapy work up	○	○	○	○	○	○	○	○	○	○	○	○
3. Improve the staff:patient ratios	○	○	○	○	○	○	○	○	○	○	○	○
4. Provide individualized information on chemotherapy side effects for patients so they are aware of what to do when unwell	○	○	○	○	○	○	○	○	○	○	○	○

5. Advise patients to check their temperature regularly to detect sepsis earlier	○	○	○	○	○	○	○	○	○	○	○	○
6. Provide information for patients and their carers on what to do when unwell e.g. card with contact numbers	○	○	○	○	○	○	○	○	○	○	○	○
7. Attach the chemotherapy prescription chart to the routine drug chart so drugs are not missed	○	○	○	○	○	○	○	○	○	○	○	○
8. Advise patients to contact hospital early in day if unwell to ensure appropriate staff available	○	○	○	○	○	○	○	○	○	○	○	○
9. Improve symptom control e.g. anti-emetics to reduce side effects and reduce delays in treatments	○	○	○	○	○	○	○	○	○	○	○	○
10. Enable staff to access patient records remotely so that on call staff are fully aware of the patient's history	○	○	○	○	○	○	○	○	○	○	○	○
11. Develop a checklist for clinicians so that important points in the history or tests are not missed	○	○	○	○	○	○	○	○	○	○	○	○
12. Ensure patients have relevant written information for community healthcare professionals to ensure that appropriate treatments are given	○	○	○	○	○	○	○	○	○	○	○	○
13. Improve drug calculation methods to reduce drug dose errors	○	○	○	○	○	○	○	○	○	○	○	○
14. Ensure other medical teams have access to the chemotherapy prescribing system and records in case patient admitted under general medical team to ensure that appropriate monitoring and treatments are provided	○	○	○	○	○	○	○	○	○	○	○	○
15. Improve monitoring of blood tests so that complications such as anaemia or electrolyte abnormalities are not missed	○	○	○	○	○	○	○	○	○	○	○	○
16. Improve communication with pharmacy about drugs and dose adjustments so that delays in drug administration do not occur	○	○	○	○	○	○	○	○	○	○	○	○
17. Improve use of GCSF when appropriate to prevent neutropenia	○	○	○	○	○	○	○	○	○	○	○	○
18. Encourage patients to undertake increased physical activity to improve physical and mental well being	○	○	○	○	○	○	○	○	○	○	○	○
19. Improve acute oncology services e.g. assessment units to fast track patients from the Emergency Department, out of hours phone numbers so that patients are seen quickly by the appropriate specialist	○	○	○	○	○	○	○	○	○	○	○	○
20. Increase use of personalised medicine approaches i.e. targeted therapies to improve outcomes and reduce side effects	○	○	○	○	○	○	○	○	○	○	○	○

Suggested solutions to chemotherapy related problems in cancer care	This solution is cost-effective				The implementation of this solution is feasible				This solution would save lives			
	Y	N	UnS	UnA	Y	N	UnS	UnA	Y	N	UnS	UnA
21. Increase the number of clinical nurse specialists to improve patient education and continuity of care	○	○	○	○	○	○	○	○	○	○	○	○
22. Develop chemotherapy prescribing clinics to focus on prescribing and ensure better communication with the chemotherapy nursing team to reduce errors	○	○	○	○	○	○	○	○	○	○	○	○

Please add any further ideas or comments that you believe are important and should be included in this study.

Y(es) : if you agree
N(o) : if you disagree
UnS(ure) : if you are aware of the problem, but unsure about the answer
UnA(ware) : if you are not sufficiently aware of the problem

Contributors to delayed diagnosis in cancer care	This patient safety threat is common				This patient safety threat leads to high rates of mortality, morbidity and incapacity				This patient safety threat affects more lower socio-economic groups or ethnic minorities				The consequences of this patient safety threat are costly to the healthcare system				This incident is amenable to a solution within 5 years			
	Y	N	UnS	UnA	Y	N	UnS	UnA	Y	N	UnS	UnA	Y	N	UnS	UnA	Y	N	UnS	UnA
1. Delays in referrals e.g. GPs not following two week referral guidelines mean that patients are diagnosed late in the course of the disease																				
2. Referrals get lost in the hospital system meaning that patients are not investigated in a timely manner																				
3. Patients present late in the course of the illness due to missed appointments leading to delay in diagnosis																				
4. Unusual symptom presentations of cancers mean that patients are not referred for investigation early																				
5. Lack of patient awareness of cancer symptoms mean that they do not attend for advice and investigation in a timely manner																				
6. Co-morbidities make it more difficult to diagnose cancer as the symptoms may be confused with those of other existing illnesses																				
7. Delays in accessing diagnostics in the community mean that patients wait longer for hospital appointments																				
8. Patient fears of the diagnosis of cancer mean that they do not seek health advice early in the course of their illness																				
9. Patients not having a GP mean that they may use other services such as the Emergency Department which are not designed to detect or diagnose cancer and hence present late																				
10. Lack of access to specialist radiology advice leads to delays in treatment plans or inappropriate treatments																				
11. GPs ignoring alarm symptoms e.g. rectal bleeding leads to delays in diagnosis																				
12. Midwives ignoring concerning symptoms e.g. breast changes during pregnancy leads to a delay in referral and diagnosis																				
13. Inappropriate referrals from primary care mean that urgent cases cannot be seen in a timely manner																				
14. Screening programs that are too selective mean that some patients are not screened and cancers are missed																				
15. GPs not having enough time mean that they do not take a full history or examine patients fully and miss cancers																				
16. Inefficient processes and bureaucracy in hospitals leads to delays in processing referrals and arranging appointments																				
17. Errors in assigning test priorities in hospital i.e. marking as routine when it should be urgent leads to inappropriate delays in the patient undergoing tests																				
18. Low risk patients are not referred for further investigations leading to a delay in diagnosis																				
19. Inability to access specialist care leads to a delayed diagnosis																				
20. Lack of communication between healthcare professionals leads to referrals not being processed in a timely manner or wrong investigations being ordered																				
21. Poor continuity of care for patients leads to symptoms being missed and delayed diagnosis																				

Please add any further ideas or comments that you believe are important and should be included in this study.

Y(es) : if you agree
N(o) : if you disagree
UnS(ure) : if you are aware of the problem, but unsure about the answer
UnA(ware) : if you are not sufficiently aware of the problem

Suggested solutions to problems that lead to delayed diagnosis in cancer care	This solution is cost-effective				The implementation of this solution is feasible				This solution would save lives			
	Y	N	UnS	UnA	Y	N	UnS	UnA	Y	N	UnS	UnA
1. Facilitate rapid referrals from primary care to hospitals	○	○	○	○	○	○	○	○	○	○	○	○
2. Encourage primary care to refer more patients and to refer earlier	○	○	○	○	○	○	○	○	○	○	○	○
3. Improve access to GPs for patients to ensure earlier diagnosis	○	○	○	○	○	○	○	○	○	○	○	○
4. Improve referral and follow up processes to ensure referrals are not lost	○	○	○	○	○	○	○	○	○	○	○	○
5. Ensure sufficient staff available to deal with referrals to ensure no delay in processing referrals	○	○	○	○	○	○	○	○	○	○	○	○
6. Encourage public awareness campaigns on common symptoms of cancer to ensure patients present early in the course of their disease	○	○	○	○	○	○	○	○	○	○	○	○
7. Improve specialist education for doctors and nurses to ensure better standards of care	○	○	○	○	○	○	○	○	○	○	○	○
8. Encourage longer consultation times to ensure a full history and examination for presenting symptoms which would lead to an earlier diagnosis	○	○	○	○	○	○	○	○	○	○	○	○
9. Increase screening for cancers to ensure earlier diagnosis	○	○	○	○	○	○	○	○	○	○	○	○
10. Improve access to diagnostics to reduce waiting time for outpatient appointments	○	○	○	○	○	○	○	○	○	○	○	○
11. Improve communication between general and oncology teams in hospitals to improve the standard of care	○	○	○	○	○	○	○	○	○	○	○	○
12. Increase sub-specialisation among cancer specialists to improve the standard of care	○	○	○	○	○	○	○	○	○	○	○	○
13. Do not give clinicians a choice in referral for certain symptoms e.g. mandate that all patients with PR bleeding are referred for a sigmoidoscopy or all breast symptoms are referred to a breast team to ensure that cancers are not missed	○	○	○	○	○	○	○	○	○	○	○	○
14. Refer people with a family history of cancer to oncology even if no symptoms to ensure cancers can be detected earlier	○	○	○	○	○	○	○	○	○	○	○	○
15. Provide prompt feedback to primary care if delayed diagnosis to encourage learning about incidents	○	○	○	○	○	○	○	○	○	○	○	○
16. Improve adherence to referral guidelines to ensure earlier diagnosis	○	○	○	○	○	○	○	○	○	○	○	○
17. If patient does not attend an appointment the hospital should continue to contact them until a response is received to ensure that they do not have a delayed diagnosis	○	○	○	○	○	○	○	○	○	○	○	○
18. Improve the quality of information in patient referrals to enable hospital clinicians to triage referrals better	○	○	○	○	○	○	○	○	○	○	○	○
19. Improve funding provided to improve services available and provide quicker access to diagnostics and specialists	○	○	○	○	○	○	○	○	○	○	○	○

Please add any further ideas or comments that you believe are important and should be included in this study.

Is there anything else for patient safety in cancer care that you would like to share with us?

Thank you very much for your time and effort.

Your contribution will help improve patient safety in cancer care.

For more information and to learn more about the outcomes of the study, please contact Dr. Josip Car at josip.car@imperial.ac.uk

Appendix S3. Framework used for analysis of contributing factors to medication safety in cancer care (London Protocol Framework)

Patient Factors: condition (complexity & seriousness), language and communication, personality and social factors

Task and Technology Factors: task design and clarity of structure, availability and use of protocols, availability and accuracy of test results, decision-making aids

Individual (staff) Factors: knowledge and skills, competence, physical and mental health

Team Factors: verbal communication, written communication, supervision and seeking help, team structure (congruence, consistency, leadership, etc.)

Work Environmental Factors: staffing levels and skills mix, workload and shift patterns design, availability and maintenance of equipment, administrative and managerial support, physical environment

Organizational and Management Factors: financial resources & constraints, organisational structure, policy, standards and goals, safety culture and priorities

Institutional Context Factors: economic and regulatory context, National Health Service executive, links with external organisations

Appendix S4. Characteristics of the respondents to the initial questionnaire

Total number of survey respondents: 40

- Oncology consultant: 15, 37.5%
- Specialist trainee (oncology, general practice, core medical training registrars and foundation doctors): 15, 37.5%
- Nurse: 6, 15%
- Cancer research scientists: 2, 5%
- Pharmacist: 2, 5%

Table S1. Ranking of all (20) cancer medication safety problems from clinicians' perspective (AEA range: 0 to 1)

Proposed solution for chemotherapy safety problems in cancer care	Breakdown point in the medication process	Contributory factor	Ranking	AEA	Responsiveness to solution	Frequency	Economic impact	Severity	Inequity
Patients with poor understanding of treatments due to language or education difficulties may miss treatments or not understand the importance of reporting side effects leading to worsening of illness	Administering / Monitoring	Patient	1	0,65	6	1	4	1	1
Insufficient attention to recognizing and managing serious psychological distress or illness due to oncological problem and treatment leads to non-compliance and/or worsening of patient's condition	Monitoring	Individual staff	2	0,53	5	4	3	8	2
Inability to obtain information on treatments given in other hospitals or by other healthcare providers e.g. palliative care team mean that the oncology team may administer inappropriate treatments or delay treatment while waiting for the information	Prescribing / Administering	Task design	3	0,54	2	5	7	5	3
Complications of central access lines inserted for chemotherapy lead to patient morbidity or delayed treatments	Administering	-	4	0,53	12	6	1	6	8
Patients have difficulty accessing acute oncology services outside of routine hours leading to delayed treatment of side effects or complications with significant negative consequences (e.g. preventable hospitalizations)	Monitoring	Organisation	5	0,56	1	12	5	4	7
Toxicity or severe allergic reactions from chemotherapy	Administering	-	6	0,55	18	2	2	2	11
Drugs may be stopped for procedures e.g. anticoagulants but not restarted leading to adverse events for patients such as thromboembolic events	Prescribing / Administering	Individual staff	7	0,53	10	8	6	3	9
Interactions between medications are not automatically highlighted meaning that inappropriate drugs may be administered together	Prescribing / Administering	Task design	8	0,49	3	3	12	9	12
Patients do not inform their oncologist of side effects meaning that the chemotherapy dose is not altered and the side effects become worse	Administering / Monitoring	Patient	9	0,46	14	13	9	7	6
Too little information on chemotherapy for patients prior to starting treatment meaning that they do not know or recognize signs of complications or serious illness and who and when to contact	Prescribing / Administering	Patient	10	0,51	4	16	14	10	4
Pharmacists spend significant amounts of time correcting errors on the chemotherapy prescribing system (e.g. ARIA) leading to delays in administration of drugs	Prescribing	Working environment	11	0,48	16	7	8	15	18

Patients attend their GP rather than oncology service for complications from chemotherapy which results in delays in treatment or inappropriate advice or treatments	Monitoring	Patient	12	0,49	9	18	20	13	5
Patients are not weighed on each cycle of chemotherapy leading to incorrect doses of chemotherapy on different cycles	Prescribing	Task design	13	0,47	7	19	15	12	10
The chemotherapy prescribing system (e.g. ARIA) does not automatically change the dates when chemotherapy is due if one dose is delayed leading to administration of chemotherapy drugs at the wrong time in the cycle	Prescribing	Task design	14	0,45	15	10	10	14	15
The chemotherapy prescribing system (e.g. ARIA) is separate from the prescribing system for other drugs leading to drug doses and drug interactions being missed	Prescribing	Individual staff	15	0,43	8	9	16	20	14
Inappropriate antibiotics are administered for sepsis	Prescribing / Administering	Individual staff	16	0,52	11	15	13	11	17
Incorrect doses of chemotherapy are administered due to inaccurate calculations of doses	Monitoring	Individual staff	17	0,44	20	14	11	16	16
Issues such as anaemia, thrombocytopenia or electrolyte disturbance from chemotherapy are not detected due to insufficient monitoring	Monitoring	Task design	18	0,54	13	20	18	17	13
The chemotherapy prescribing system (e.g. ARIA) does not automatically carry forward dose adjustments in regimens leading to inappropriate dosing of chemotherapy drugs	Prescribing	Task design	19	0,42	19	11	17	18	20
The chemotherapy prescribing system (e.g. ARIA) is difficult to use leading to drug dosing and timing errors	Prescribing	Task design	20	0,43	17	17	19	19	19

AEA –average expert agreement

Table S2. Ranking of all (22) solutions to cancer medication safety problems from clinicians' perspective (AEA range: 0 to 1)

Proposed solution for chemotherapy safety problems in cancer care	Breakdown point in the medication process	Related defence barrier	Ranking	AEA	Cost-effectiveness	Feasibility	Saving Lives
Provide information for patients and their carers on what to do when unwell e.g. card with contact numbers	Prescribing/Administering	Task design/Patient	1	0,92	4	1	3
All patients should receive an appropriate pre-chemotherapy work up	Administering	Task design	2	0,88	1	3	4
Improve training of staff	Prescribing, transcribing, dispensing, administering, monitoring	Working environment	3	0,88	6	6	1
Develop a checklist for clinicians so that important points in the history or tests are not missed	Prescribing	Task design	4	0,83	5	4	8
Ensure patients have relevant written information for community healthcare professionals to ensure that appropriate treatments are given	Prescribing/Administering	Task design/Patient	5	0,88	3	10	5
Enable staff to access patient records remotely so that on call staff are fully aware of the patient's history	Prescribing/Administering/Monitoring	Task design	6	0,83	11	2	7
Improve the staff:patient ratios	Prescribing, transcribing, dispensing, administering, monitoring	Working environment	7	0,83	17	7	2
Improve communication with pharmacy about drugs and dose adjustments so that delays in drug administration do not occur	Transcribing	Team	8	0,85	10	5	17
Advise patients to check their temperature regularly to detect sepsis earlier	Monitoring	Task design/Patient	9	0,83	8	8	15
Attach the chemotherapy prescription chart to the routine drug chart so drugs are not missed	Prescribing	Task design	10	0,82	2	9	20
Advise patients to contact hospital early in day if unwell to ensure appropriate staff available	Monitoring	Task design/Patient	11	0,82	7	15	12
Improve acute oncology services e.g. assessment units to fast track patients from the Emergency Department, out of hours phone numbers so that patients are seen quickly by the appropriate specialist	Prescribing/Monitoring	Organization	12	0,77	16	13	6
Improve symptom control e.g. anti-emetics to reduce side effects and reduce delays in treatments	Monitoring	Task design	13	0,77	9	19	16
Increase the number of clinical nurse specialists to improve patient education and continuity of care	Monitoring	Working environment	14	0,77	19	14	10
Encourage patients to undertake increased physical activity to improve physical and mental well being	Monitoring	Task design/Patient	15	0,73	18	12	19

Ensure other medical teams have access to the chemotherapy prescribing system and records in case patient admitted under general medical team to ensure that appropriate monitoring and treatments are provided	Monitoring	Team	16	0,73	12	21	13
Provide individualized information on chemotherapy side effects for patients so they are aware of what to do when unwell	Prescribing/Administering	Task design	17	0,73	14	20	14
Improve use of GCSF when appropriate to prevent neutropenia	Prescribing	Task design	18	0,67	21	11	18
Improve monitoring of blood tests so that complications such as anaemia or electrolyte abnormalities are not missed	Monitoring	Task design	19	0,72	13	17	22
Improve drug calculation methods to reduce drug dose errors	Prescribing	Task design	20	0,73	15	16	21
Develop chemotherapy prescribing clinics to focus on prescribing and ensure better communication with the chemotherapy nursing team to reduce errors	Prescribing	Organization	21	0,70	20	22	11
Increase use of personalised medicine approaches i.e. targeted therapies to improve outcomes and reduce side effects	Prescribing	Task design	22	0,68	22	18	9

AEA –average expert agreement; GCSF - Granulocyte-colony stimulating factor
